# Supplementary material for: Chromatin accessibility landscape of stromal subpopulations reveals distinct metabolic and inflammatory features of porcine subcutaneous and visceral adipose tissue
Source: PeerJ. 2022 May 24;10:e13250. doi: 10.7717/peerj.13250 (PMC9138157; doi:10.7717/peerj.13250)
Supplement: Supplemental Information 8 [file peerj-10-13250-s008.docx]

| **Gene symbol** | **Functions** |
| --- | --- |
| ACSS3 | ACSS3 (Acyl-CoA Synthetase Short Chain Family Member 3) is a Protein Coding gene. Diseases associated with ACSS3 include Mosaic Variegated Aneuploidy Syndrome. Among its related pathways are ethanol degradation II and Ketone body metabolism. Gene Ontology (GO) annotations related to this gene include acetate-CoA ligase activity. An important paralog of this gene is ACSS1. |
| ARHGAP26 | ARHGAP26 (Rho GTPase Activating Protein 26) is a Protein Coding gene. Diseases associated with ARHGAP26 include Juvenile Myelomonocytic Leukemia and Leukemia. Among its related pathways are Integrin Pathway and Signaling by GPCR. GO annotations related to this gene include GTPase activator activity and phospholipid binding. An important paralog of this gene is ARHGAP10. |
| ARHGEF3 | ARHGEF3 (Rho Guanine Nucleotide Exchange Factor 3) is a Protein Coding gene. Diseases associated with ARHGEF3 include Methotrexate-Associated Lymphoproliferation and Osteoporosis. Among its related pathways are Interferon Pathway and NgR-p75(NTR)-Mediated Signaling. GO annotations related to this gene include guanyl-nucleotide exchange factor activity. An important paralog of this gene is NET1 |
| BANP | BANP (BTG3 Associated Nuclear Protein) is a Protein Coding gene. Diseases associated with BANP include Brittle Cornea Syndrome and Keratoconus. Among its related pathways are Regulation of TP53 Activity and Gene Expression. GO annotations related to this gene include p53 binding. |
| COL5A1 | COL5A1 (Collagen Type V Alpha 1 Chain) is a Protein Coding gene. Diseases associated with COL5A1 include Ehlers-Danlos Syndrome, Classic Type, and Fibromuscular Dysplasia, Multifocal. Among its related pathways are Integrin Pathway and ERK Signaling. GO annotations related to this gene include heparin binding and extracellular matrix structural constituent. An important paralog of this gene is COL11A1. |
| CRYM | CRYM (Crystallin Mu) is a Protein Coding gene. Diseases associated with CRYM include Deafness, Autosomal Dominant 40 and Autosomal Dominant Non-Syndromic Sensorineural Deafness Type Dfna. Among its related pathways are lysine degradation II (pipecolate pathway) and Histidine, lysine, phenylalanine, tyrosine, proline and tryptophan catabolism. GO annotations related to this gene include protein homodimerization activity and NADP binding. |
| EBF3 | EBF3 (EBF Transcription Factor 3) is a Protein Coding gene. Diseases associated with EBF3 include Hypotonia, Ataxia, And Delayed Development Syndrome and Neurogenic Bladder. Among its related pathways are Differentiation of white and brown adipocyte. GO annotations related to this gene include protein dimerization activity. An important paralog of this gene is EBF1. |
| FKBP5 | FKBP5 (FKBP Prolyl Isomerase 5) is a Protein Coding gene. Diseases associated with FKBP5 include Major Depressive Disorder and Asthma. Among its related pathways are PI3K / Akt Signaling and MECP2 and Associated Rett Syndrome. GO annotations related to this gene include peptidyl-prolyl cis-trans isomerase activity and FK506 binding. An important paralog of this gene is FKBP4. |
| GTDC1 | GTDC1 (Glycosyltransferase Like Domain Containing 1) is a Protein Coding gene. Diseases associated with GTDC1 include Mowat-Wilson Syndrome and Meier-Gorlin Syndrome. GO annotations related to this gene include glycosyltransferase activity and transferase activity. |
| HPGD | HPGD (15-Hydroxyprostaglandin Dehydrogenase) is a Protein Coding gene. This gene encodes a member of the short-chain nonmetalloenzyme alcohol dehydrogenase protein family. The encoded enzyme is responsible for the metabolism of prostaglandins, which function in a variety of physiologic and cellular processes such as inflammation.Diseases associated with HPGD include Digital Clubbing, Isolated Congenital and Hypertrophic Osteoarthropathy, Primary, Autosomal Recessive. Among its related pathways are Arachidonic acid metabolism and Metabolism. GO annotations related to this gene include protein homodimerization activity and NAD binding. |
| LDB2 | LDB2 (LIM Domain Binding 2) is a Protein Coding gene. Diseases associated with LDB2 include Retinal Detachment and Nail-Patella Syndrome. Among its related pathways are Ectoderm Differentiation. GO annotations related to this gene include enzyme binding and obsolete transcription factor activity, transcription factor binding. An important paralog of this gene is LDB1. |
| MAML2 | MAML2 (Mastermind Like Transcriptional Coactivator 2) is a Protein Coding gene. Diseases associated with MAML2 include Hidradenoma and Mucoepidermoid Carcinoma. Among its related pathways are Signaling by NOTCH1 and Notch Signaling Pathway. GO annotations related to this gene include transcription coactivator activity. An important paralog of this gene is MAML3 |
| NR1D1 | NR1D1 (Nuclear Receptor Subfamily 1 Group D Member 1) is a Protein Coding gene. Diseases associated with NR1D1 include Hypothyroidism, Congenital, Nongoitrous, and Rem Sleep Behavior Disorder. Among its related pathways are Circadian Clock in Mammals and Regulation of lipid metabolism by Peroxisome proliferator-activated receptor alpha (PPARalpha). GO annotations related to this gene include DNA-binding transcription factor activity and RNA polymerase II cis-regulatory region sequence-specific DNA binding. An important paralog of this gene is NR1D2. |
| PDE4B | PDE4B (Phosphodiesterase 4B) is a Protein Coding gene. Diseases associated with PDE4B include Schizophrenia and Schizophrenia. Among its related pathways are Sweet Taste Signaling and Myometrial Relaxation and Contraction Pathways. GO annotations related to this gene include transmembrane transporter binding and cAMP binding. An important paralog of this gene is PDE4D. |
| PLD1 | PLD1 (Phospholipase D1) is a Protein Coding gene. Diseases associated with PLD1 include Cardiac Valvular Defect, Developmental and Coffin-Lowry Syndrome. Among its related pathways are Sweet Taste Signaling and Factors and pathways affecting insulin-like growth factor (IGF1)-Akt signaling. GO annotations related to this gene include phosphatidylinositol binding and N-acylphosphatidylethanolamine-specific phospholipase D activity. An important paralog of this gene is PLD2. |
| RAPGEF5 | RAPGEF5 (Rap Guanine Nucleotide Exchange Factor 5) is a Protein Coding gene. Diseases associated with RAPGEF5 include Familial Isolated Hypoparathyroidism. Among its related pathways are G-protein signaling_Cross-talk between Ras-family GTPases and G-protein signaling Regulation of p38 and JNK signaling mediated by G-proteins. GO annotations related to this gene include guanyl-nucleotide exchange factor activity and guanyl-nucleotide exchange factor activity. An important paralog of this gene is RAPGEF4 |
| RYR2 | RYR2 (Ryanodine Receptor 2) is a Protein Coding gene. Diseases associated with RYR2 include Ventricular Tachycardia, Catecholaminergic Polymorphic, With Or Without Atrial Dysfunction And/Or Dilated Cardiomyopathy and Arrhythmogenic Right Ventricular Dysplasia, Familial. Among its related pathways are Cell-type Dependent Selectivity of CCK2R Signaling and Ion channel transport. GO annotations related to this gene include calcium ion binding and protein kinase binding. An important paralog of this gene is RYR3. |
| SPIDR | SPIDR (Scaffold Protein Involved In DNA Repair) is a Protein Coding gene. Diseases associated with SPIDR include Genetic Non-Acquired Premature Ovarian Failure and ,Xx Sex Reversal. Among its related pathways are Resolution of D-Loop Structures and Homology Directed Repair. |
| STAT3 | STAT3 (Signal Transducer And Activator Of Transcription 3) is a Protein Coding gene. This protein is activated through phosphorylation in response to various cytokines and growth factors including IFNs, EGF, IL5, IL6, HGF, LIF and BMP2. This protein mediates the expression of a variety of genes in response to cell stimuli, and thus plays a key role in many cellular processes such as cell growth and apoptosis. The small GTPase Rac1 has been shown to bind and regulate the activity of this protein. PIAS3 protein is a specific inhibitor of this protein. This gene also plays a role in regulating host response to viral and bacterial infections. Mutations in this gene are associated with infantile-onset multisystem autoimmune disease and hyper-immunoglobulin E syndrome. Diseases associated with STAT3 include Hyper-Ige Recurrent Infection Syndrome, Autosomal Dominant and Autoimmune Disease, Multisystem, Infantile-Onset. Among its related pathways are RET signaling and Signaling by PTK6. GO annotations related to this gene include DNA-binding transcription factor activity and sequence-specific DNA binding. An important paralog of this gene is STAT1. |
