# Supplementary figures and images for: Chromatin accessibility landscape of stromal subpopulations reveals distinct metabolic and inflammatory features of porcine subcutaneous and visceral adipose tissue
Source: PeerJ. 2022 May 24;10:e13250. doi: 10.7717/peerj.13250 (PMC9138157; doi:10.7717/peerj.13250)

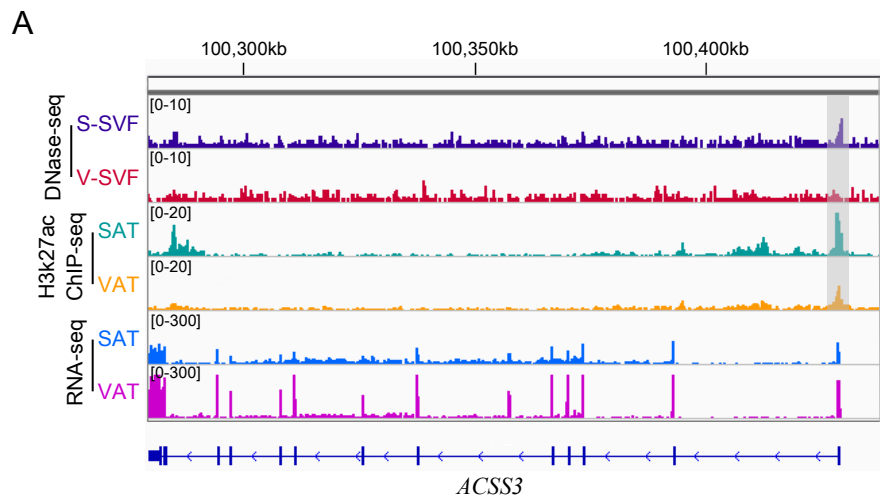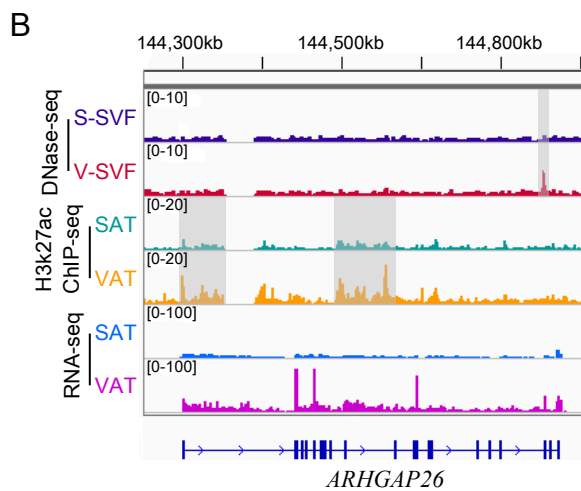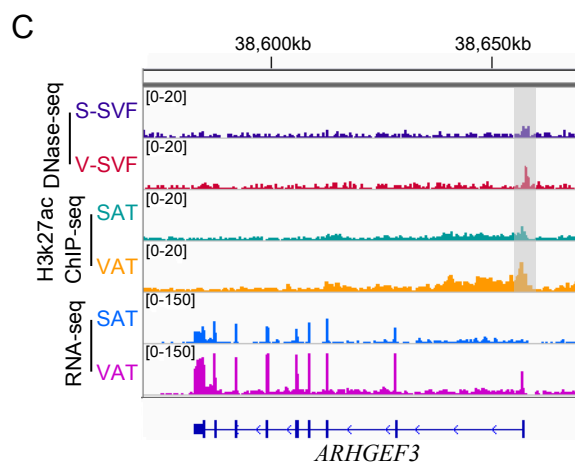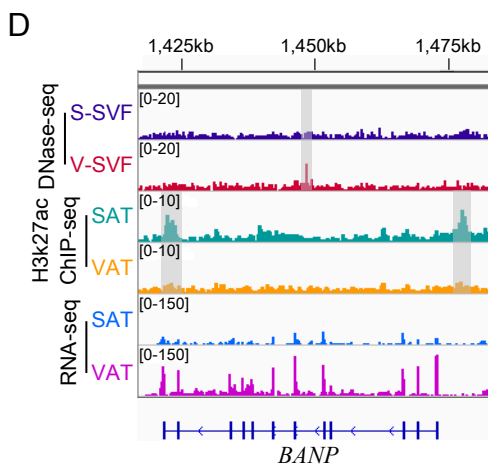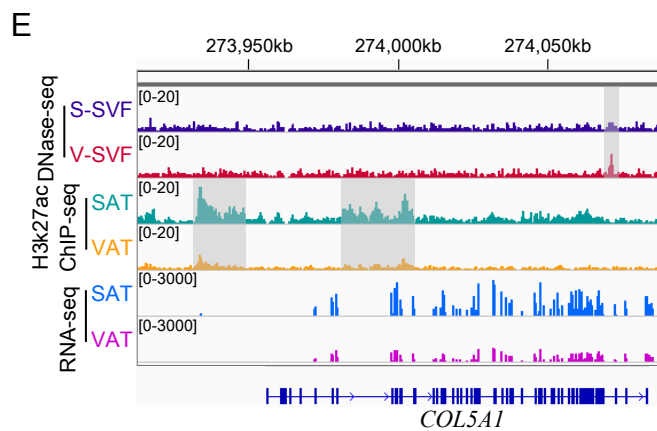

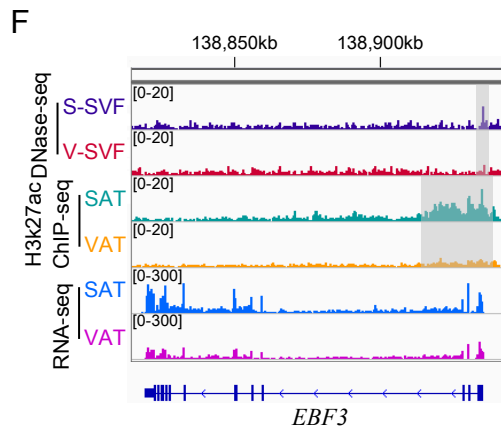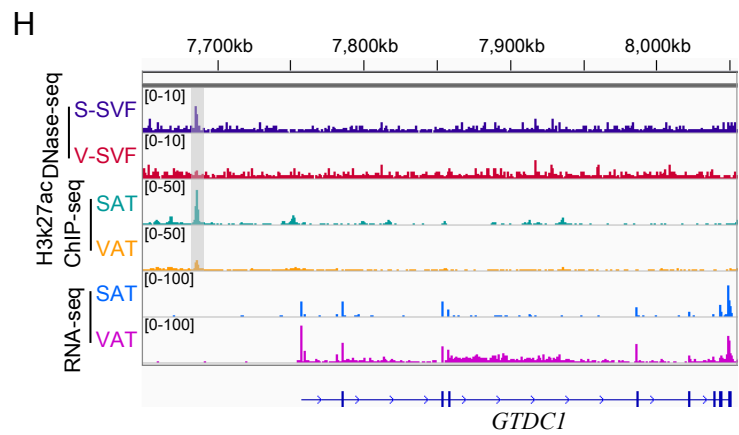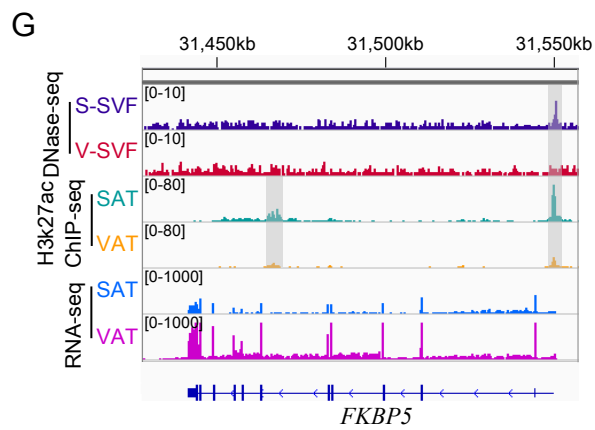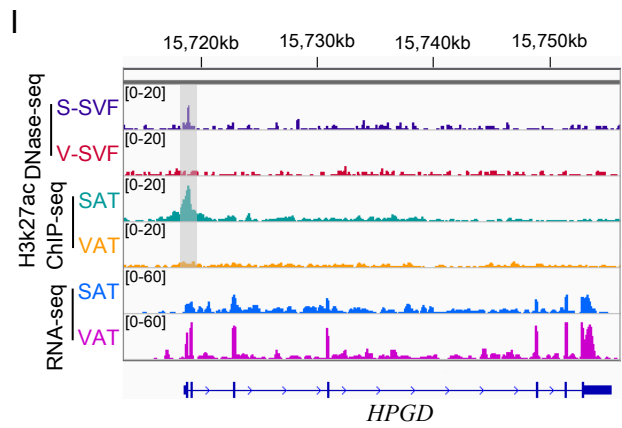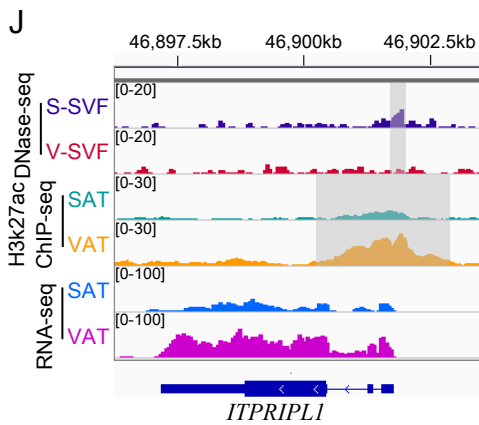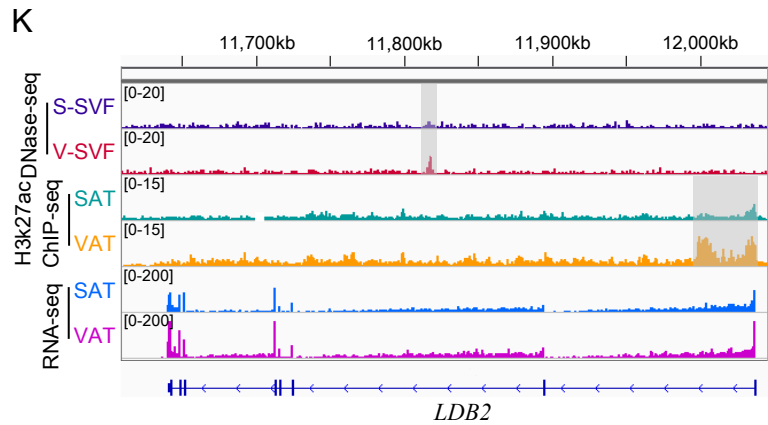

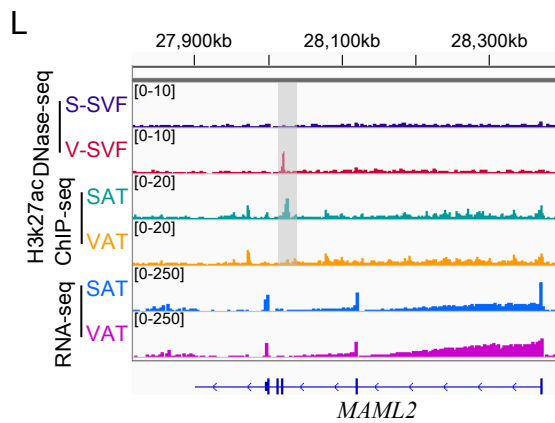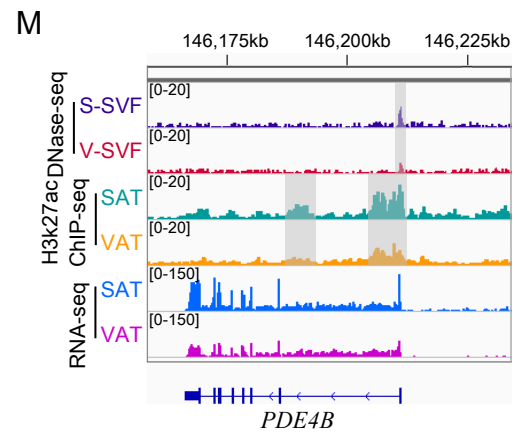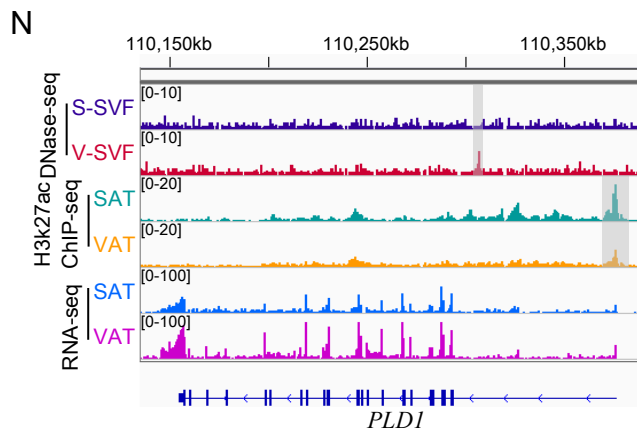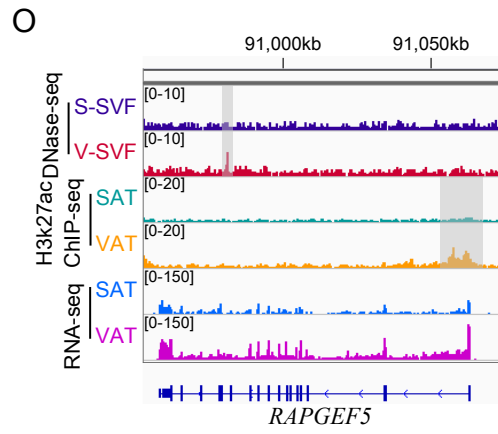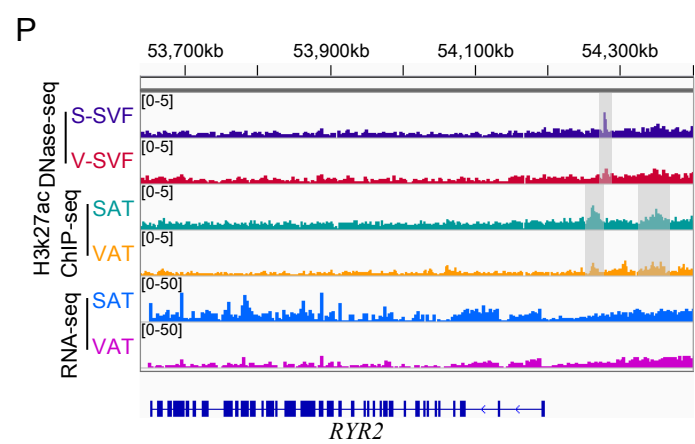

Q

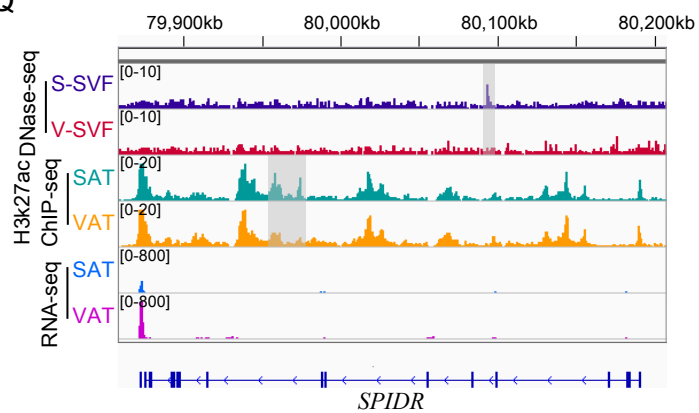

R

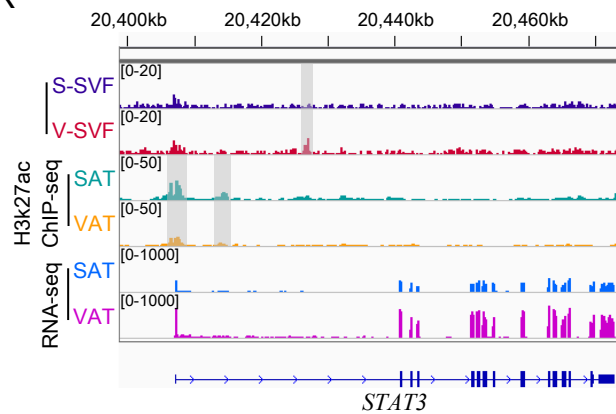

Supplement: Supplemental Information 9 — Genome browser showing DNase-seq signals, H3K27ac ChIP-seq signals, and RNA-seq expression profiles around the 18 genes in Fig. 4C. Gray boxes indicate DHSs and ChIP-seq peaks. [file peerj-10-13250-s009.pdf]
